# Development and Initial Validation of a Rock Climbing Craving Questionnaire (RCCQ)

**DOI:** 10.3389/fpsyg.2018.00204

**Published:** 2018-02-22

**Authors:** Gareth Roderique-Davies, Robert M. Heirene, Stephen Mellalieu, David A. Shearer

**Affiliations:** ^1^School of Psychology and Therapeutic Studies, University of South Wales, Pontypridd, United Kingdom; ^2^Cardiff School of Sport, Cardiff Metropolitan University, Cardiff, United Kingdom

**Keywords:** extreme sport, climbing, craving, reinforcement, withdrawal

## Abstract

Conceptual similarities have been identified between experiences of extreme sports athletes and those with drug and behavioral addictions. Evidence suggests rock climbers experience craving and other withdrawal-like states when abstinent from their sport. However, no studies have attempted to quantitatively measure the craving experienced by participants of any extreme sports. Such a measure could allow a greater understanding of the craving experienced by extreme sports athletes and a comparison of these across sports (e.g., surfing) and activities (e.g., drug-use). Therefore, using validated craving measures as a template, the aim of the two studies outlined here was to design and preliminarily validate a subjective multidimensional inventory that could be used to measure craving in the sports of rock-climbing and mountaineering (“RCCQ”). The aim of the first study was to investigate the factor structure of a preliminary measure of craving. Climbers (*n* = 407) completed the RCCQ. A 3-factor model explained 53.65% of the total variance in item scores. All three factors comprised five items each, which were conceptually labeled as “urge to climb” “negative reinforcement” and “positive reinforcement.” The aim of the second study was to validate the 15-item 3-factor RCCQ resulting from Study 1 using confirmatory factor analysis (CFA). Climbers (*n* = 254) completed the questionnaire under a climbing-related cue condition or a cue-neutral condition. CFA revealed a good model fit and that all individual parameter estimates were significant and standard errors were within reasonable limits once item 13 was removed from Factor 1. Study 1 supports the multi-dimensional nature of rock climbing craving and shows parallels with substance-related craving in reflecting intention and positive (desire) and negative (withdrawal) reinforcement. Study 2 confirms this factor structure and gives initial validation to the measure with evidence that these factors are sensitive to cue exposure. Given the preliminary nature of the data, any practical implications are tentative. However, if as shown here, craving for climbing (and potentially other extreme sports) is similar to that experienced by drug-users and addicts, there is the potential that climbing and other extreme sports could be used as a replacement therapy for drug users.

## Introduction

A number of conceptual similarities have been identified between the psychological and behavioral experiences of extreme sports athletes and those with drug and behavioral addictions. Buckley (2015) recently provided a systematic exposition of the similarities between the two populations based on >30,000 hours of ethnographic observation and 160 interviews with extreme sports athletes, arguing parallels between the preoccupation with and prioritization of the activity, mood modification and emotional reward seeking, the development of tolerance and the experience of withdrawal symptoms. Buckley’s comparisons are corroborated by extant studies of extreme sports athletes’ experiences. For example, Franken et al. (2006) found that during periods of abstinence skydivers experienced anhedonia, a negative mood state often observed in withdrawing drug-users. Strong cravings or urges for participation have also been observed in skydivers (Price and Bundesen, 2005) and, more recently, Heirene et al. (2016) found evidence to suggest rock climbers also experience withdrawal states when abstinent from their sport. When not climbing, the participants reported urges or cravings for their sport, negative affective experiences and anhedonic symptoms.

While drug-users and extreme sports exponents may experience similar psychological states, their behaviors are distinguished on the grounds of legality, social acceptance and arguably health promotion. Thus, the impact of excessive engagement in extreme sports may be of less detriment to a person’s personal, social and professional life compared with excessive drug-use. Indeed, caution must be taken to avoid pathologizing all avid extreme sports athletes as “addicts” who must be treated (cf., Billieux et al., 2015). Extreme sports provide a socially acceptable medium through which individuals can exercise, develop skills, satisfy sensation seeking traits (Diehm and Armatas, 2004), become an agent of their emotions (Woodman et al., 2010), and experience freedom (Brymer and Schweitzer, 2013), challenge, and new environments (Kerr and Mackenzie, 2012). Nonetheless, the positive outcomes associated with extreme sports participation do not reduce the validity of the observed parallels between extreme sports athletes and drug-users, rather they imply the similarities may have valuable implications for the study and treatment of addiction.

If avid extreme sports athletes are addicted to some degree, they may provide an easily accessible and potentially willing population for the study of addiction (Buckley, 2015). Further research is also warranted on the study of extreme sports addiction and its similarities with substance addictions, given the recent impetus for using extreme sports as an intervention for mental health problems (Luttenberger et al., 2015; Clough et al., 2016). However, currently no measures of addiction or allied constructs have been developed and validated for use with extreme sports athletes, restricting its study within this population. Consequently, in the present study we aimed to develop and validate a craving measure applicable to extreme sports athletes, given the central role played by craving in drug-addiction maintenance and relapse (Tiffany and Wray, 2012). Craving was also central to rock climbers’ experiences in Heirene et al. (2016), where it was conceptualized as a strong, overarching need to participate, with reports of urges and compulsions comparable to that observed in those with an addiction. Such a measure could allow a greater understanding of the degree of craving experienced by extreme sports athletes and a comparison of these across sports (e.g., surfing) and activities (e.g., drug-use), as well as a greater understanding of the association between the degree of craving and other states (e.g., withdrawal symptoms) and behaviors (e.g., over training, excessive risk-taking).

In the psychopharmacological literature craving is defined as a subjective motivational state in which an individual experiences an intense desire to engage in drug-taking (UNDCP and WHO, 1992). Craving is believed to be “at the heart” of addiction and factors including abstinence (Tiffany, 1990), mood states (Shen et al., 2012) and cue-exposure (Kreusch et al., 2017) are known to mediate drug-craving. A number of conceptual similarities between the craving experienced by drug users and that by extreme sports athletes have been identified. For example, rock climbers in Heirene et al.’s (2016) study suggested exposure to climbing related stimuli increased their craving. Additionally, both rock climbers in Heirene et al. (2016) and skydivers in Price and Bundesen (2005) suggested their urges were often underpinned by a motive to remove negative psychological states or “self-medicate.”

To date, craving has predominantly been measured using the original multidimensional Questionnaire of Smoking Urges (QSU; Tiffany and Drobes, 1991) and its many derivatives applied to other substances, including alcohol (Clark, 1994; Singleton et al., 2004) cocaine (Tiffany et al., 1993), amphetamine (James et al., 2004), and caffeine (West and Roderique-Davies, 2007). The original questionnaire consisted of 32 items and 2 factors, labeled as positive reinforcement and negative reinforcement. However, since then, the other derivatives of the QSU have found that three factors best fit the data. For example, during the development of the Alcohol Craving Questionnaire (ACQ; Singleton et al., 2004) and the Desires for Alcohol Questionnaire (DAQ; Clark, 1994), both sets of authors found a third factor representing “No desire to drink” and “Mild desires and intentions,” respectively.

No studies have yet attempted to quantitatively measure the craving experienced by participants of extreme sports. Consequently, there are no validated measures suitable for this purpose. Therefore, using the original QSU as a template, the aim of the two studies outlined here was to design and preliminarily validate a subjective multidimensional inventory which could be used to measure craving in the sports of rock-climbing and mountaineering. Rock-climbers and mountaineers were sampled for several reasons. First, findings from Heirene et al. (2016) study suggest climbers of different ability levels experience craving for their sport. Second, they represent ‘true’ extreme sports, where serious mistakes of judgment can have serious consequences (i.e., severe injury or death). Finally, within the United Kingdom, rock climbing and mountaineering have both high participation rates and broad appeal compared to other niche extreme sports (e.g., BASE-jumping). For example, UKclimbing.com, the most popular climbing website and forum in the United Kingdom, has over 48,000 users ranging in age from under 21 to over 50, providing a rich source of data.

## Study 1

The aim of the first study was to investigate the factor structure of a preliminary measure of extreme sports craving using an adapted version of the original 32 item QSU. Although current trends suggest the use of confirmatory factor analysis (CFA) is preferable for testing hypotheses about the structure of latent variables (Biddle et al., 2001), given the novel and exploratory nature of the research, and the precedent in analysis methods used by previous derivatives of the QSU (e.g., James et al., 2004), it was deemed appropriate in this instance to use exploratory factor analysis as a first stage (Field, 2013, p. 666). Therefore, while it was expected that the data would be similar to recent versions of the QSU (e.g., West and Roderique-Davies, 2007), no specific hypotheses were made about the factor structure.

### Questionnaire Development

We based the Rock Climbing Craving Questionnaire (RCCQ) on previously validated, substance-specific, multi-dimensional craving questionnaires that utilized the Questionnaire of Smoking Urges (QSU, Tiffany and Drobes, 1991) as their foundation (see Davies et al., 2000, Cigarettes; James et al., 2004, Amphetamines; Love et al., 1998, Alcohol; Tiffany et al., 1993, Cocaine; West and Roderique-Davies, 2007, Caffeine). Each of these questionnaires conceptualizes “craving” in terms of relief of withdrawal or negative affect, desires and intentions, and anticipation of positive outcomes. Initially, the 38 items from the most recent of these questionnaires (caffeine craving questionnaire; West and Roderique-Davies, 2007) were transformed into climbing relevant items, such that the word “climbing” replaced “caffeine” where appropriate. In addition, some items were changed from the passive sense (e.g., *to have some caffeine*) to the active sense (e.g., *to go climbing*). Three items from the Caffeine Craving Questionnaire were inappropriate in this context (e.g., *I would feel more nauseous if I was having caffeine*) and were removed leaving 35 items.

A preliminary version of the RCCQ was subjected to peer-appraisal and administered to 10 volunteers. This suggested sufficient levels of congruence between the researchers’ and participants’ meaning of each item. Each item was presented on a separate page, which required a response before answering the next item. Participants were asked to report their level of agreement with each statement by responding on a Likert-type scale to each item, anchored by 1 (*strongly disagree*) and 7 (*strongly agree*; Tiffany and Drobes, 1991), with 12 of the items being reverse-keyed.

### Participants

A total of 407 individuals (mean age: 35.37, *SD*: 13.75, 326 male, 81 female) completed the RCCQ. Climbers from 20 different countries across five continents participated, and a variety of primary climbing disciplines were reported, comprising traditional climbing (*n* = 175; 43%), indoor climbing (*n* = 100; 24.6%), sport climbing (*n* = 70; 17.2%), bouldering (*n* = 49; 12%), alpine climbing (*n* = 10; 2.5%), mixed climbing (*n* = 2; 0.5%) and ice climbing (*n* = 1; 0.2%). A prerequisite to participation was that all climbers had a minimum of 6 months of climbing experience. The length of participants’ climbing experience varied, with 29 having 6 months to 1 year’s experience, 91 having 1–3 years, 75 3–6 years, 43 6–9 years, 28 9–12 years and 141 with more than 12 years’ experience. Frequency of climbing varied from 3 or more times per week to at least once per year, with most stating they climbed at least once a week (*n* = 178) or 3 or more times per week (*n* = 134). The length of time since participants’ last climb varied from less than 1 day to 400 days, with a mode of 1 day. Climbing ability varied significantly, from novice (best worked grade: traditional climbing: VS, sport climbing: Fr5) to elite level (best worked grade: traditional climbing: E9 7a, sport climbing: Fr8b; bouldering: V9).

### Procedures

Two forms of participant recruitment and data collection were implemented. In one method, rock climbers and mountaineers were recruited from two-UK-Based internet climbing forums. Specifically, a premier post (a forum posting which remains at the top of the page) was purchased which outlined the details of the study and asked users to participate. Participants completed the questionnaire on-line via Surveymonkey. To control for multiple submissions each respondent’s IP address was logged and subsequently blocked following questionnaire completion. In the second method, participants were sampled from indoor climbing gyms in South Wales. Participants recruited via this method completed the survey in paper format or online using a laptop provided by the research team.

### Ethics Statement

Ethics approval was sought from the first author’s School Ethics Committee prior to commencing both studies. This study was carried out in accordance with the recommendations of BPS code of Human Research Ethics with written informed consent from all participants. All participants gave written informed consent in accordance with the Declaration of Helsinki. The protocol was approved by the University of South Wales School of Psychology and Therapeutic Studies Ethics subcommittee.

### Statistical Analysis

Of the original 407 cases only 368 cases were included in the principal component analysis as cases with missing values were deleted listwise. Responses to reverse-keyed items were inverted before the main analysis. Data was pre-screened for multicollinearity by examining inter-item correlations for values above *r* = 0.9; none were observed. Kaiser-Meyer-Olgin measure of sampling adequacy was 0.93 indicating the sample size was large enough and Bartlett’s test of sphericity was significant (*p* < 0.001) indicating the original correlation matrix was an identity matrix as required. Principal component analysis was performed using the oblique (Oblimin) method (Hendrickson and White, 1964) as this was expected to produce the ‘cleanest’ factors (Love et al., 1998; James et al., 2004). The initial PCA requested was ‘unforced,’ with subsequent iterations ‘forced’ in order to identify the most parsimonious model. In the initial unforced PCA only factors with eigenvalues greater than one were retained and subjected to scrutiny regarding their item content (Tiffany and Drobes, 1991; Davies et al., 2000; James et al., 2004). Items were considered meaningful only if they loaded at higher than 0.4 on a given factor, and that this loading was at least 0.2 greater the item’s next highest loading on another factor (Tiffany and Drobes, 1991; Davies et al., 2000; James et al., 2004). For the forced PCA that followed, eigenvalue criteria were moot, however, the method for scrutinizing items was the same.

### Results

The first iteration of the PCA whereby all items were free to load onto any number of factors resulted in a 5-factor model, with Eigenvalues of 14.55, 2.36, 1.87, 1.35, and 1.13. The total variance explained by this model was 60.71%, with variance values for each Factor (1–5) of 41.57, 6.70, 5.36, 3.86, and 3.21. Factor loading for each item revealed that Factor 1 contained seven items, Factor 2 contained six items, Factor 3 contained five items, Factor 4 contained four items and Factor 5 contained only two items. Although item loadings made conceptual sense (conceptual labels of “positive reinforcement,” “negative reinforcement,” “mood and well-being,” “temporal plans,” and “extreme events” were applied in order) a decision was made to test a four-factor model due to the small number of items within the 5th factor.

The four-factor model explained a total 57.5% of the variance, with variance values for each Factor (1–4) of, 41.58, 6.70, 5.36, and 3.86. Factor loading for each item revealed that Factors 1 and 2 contained six items each, Factor 3 contained three items, and Factor 4 contained two items. As before, loading made some conceptual sense, however, Factors 2 and 3 seemed to both measure negative reinforcement, while the two items for Factor 4 (labeled “urge to climb”) were insufficient for a reasonable measure of internal reliability. Therefore, the decision was made to force one final model that contained only three factors.

The three-factor model explained a total of 53.65% of the total variance in item scores, with values for each Factors (1–3) of 41.57, 6.70, and 5.36. All three factors comprised five items each, which were conceptually labeled as “urge to climb,” “negative reinforcement,” and “positive reinforcement” (**Table 1**). Even though this model explained the least amount of variance of the three models tested here, the model appeared to have the strongest face validity, made conceptual sense, had an equal balance of items per factor and was the most parsimonious model tested. Therefore, the decision was made to use this model as the basis for the next stage of the scale validation.

**Table 1:** Rock climbing craving questionnaire (RCCQ) items factor loadings.

	Factor loading		
RCCQ Item	Factor 1	Factor 2	Factor 3
**Factor 1 – Urge to climb**	0.679		
*5. I will go climbing as soon as I get the chance*	0.532		
*10. Starting now, I could go without climbing for a long time*	0.802		
*13. All I want to do right now is go climbing*	0.759		
*24. I am going to go climbing as soon as possible*	0.479		
*31. Right now, I am not making any plans to go climbing*	0.679		
**Factor 2 – Negative reinforcement**		0.727	
*7. Climbing would make me less depressed*		0.633	
*8. Climbing would not help me calm down*		0.808	
*23. My head would be clearer right now if I could go climbing*		0.784	
*28. If I were climbing now, I could think more clearly*		0.781	
*32. It would feel as if the bad things in my life had completely disappeared if I went climbing now*		0.727	
**Factor 3 – Positive reinforcement**			0.663
*1. Climbing would make me feel very good right now*			0.604
*11. Going climbing would not be pleasant*			0.653
*14. Climbing right now would invigorate me*			0.752
*21. I would not enjoy climbing right now*			0.677
*26. Going climbing would not be very satisfying now*			0.663

## Study 2

The primary aim of the second study was to validate the 15 item 3 factor RCCQ resulting from Study 1 using CFA. The secondary aim was to investigate predictive validity of the RCCQ to two factors known to influence acute craving: abstinence and climbing-related cue exposure.

### Participants

In total, 254 climbers (mean age: 31.6, *SD*: 10.1, 209 male, 70 female) took part in the study. While a second process of data collection was implemented for this study following the analysis of findings from Study 1, it is possible that some of the climbers who took part in the first study also participated in Study 2. The same participant recruitment and data collection methods used for Study 1 were replicated for this study, with one exception. Approximately half the participant (*n* = 125) completed the questionnaire while being simultaneously presented with climbing related cues in the form of visual media depicting either climbing itself or climbing related equipment (e.g., a rope and harness). A new picture was presented next to each question in the craving measure as the online survey was completed. Similar variance to the first study’s sample was observed in participants’ climbing discipline, duration, frequency, days since last time and ability.

### Statistical Analysis

Confirmatory factor analysis using EQS 6.01 software (Bentler, 2005) was used to assess the fit of the data from this new sample to the proposed 15-item scale identified by the PCA in Study 1. First, data was screened for multivariate normality using Mardia’s (1970, 1974). Normalized Coefficient whereby values greater than 5 indicated non-normal data (Bentler, 2005). In this instance Mardia’s normalized value was 17.73 and therefore all CFA models were run using the ROBUST statistic method available in the EQS software, which runs the Satorra–Bentler Chi Square (S–B χ^2^: Satorra and Bentler, 1994) and calculates robust standard errors (Bentler and Dijkstra, 1985). The Maximum Likelihood (ML) estimation process was employed as research has indicated that parameter estimates remain valid even when the data are non-normal (Satorra and Bentler, 1994).

Confirmatory factor analysis models were assessed both in terms of overall model fit and for each individual parameter estimate. Fit indices were chosen to indicate model fit, model comparison, and model parsimony and included Root Mean Square Error of Approximation (RMSEA: Steiger and Lind, 1980), the Comparative Fit index (CFI: Bentler and Bonett, 1980), the Non-Normed Fit Index (NNFI: Tucker and Lewis, 1973) and the McDonald Fit Index (MFI: McDonald, 1989). The criterion scores used to indicate a good fit to the proposed model were: <0.06 for RMSEA, >0.90–0.95 for CFI and NNFI, and >0.89 for the MFI (Hu and Bentler, 1999). Lagrange Multiplier tests (LM χ^2^) were used to test the viability of specific parameters in the proposed model (Byrne, 2008), where a significant LM χ^2^ indicates factor cross loadings, error covariance, and consequently model misspecification. Misspecification is indicated when a parameter set shows incremental univariate χ^2^ values substantially greater than the other parameter sets (see Byrne, 2008, p. 111). This information can then be used to guide further specification searches before finalizing the model.

ANCOVA and MANCOVA was used, respectively, to examine the difference in total craving scores and each of the three craving factors between those who completed the questionnaire while viewing climbing related cues and those who did not. The covariates of ‘climbing frequency,’ ‘day since last climb,’ and ‘total climbing experience’ were added to both models. For the MANCOVA a follow-up principal component analysis was used to examine the nature of significant differences between the two conditions (Field, 2013). Finally, four separate simple regressions were performed to examine whether the number of days since participants last climbed predicted either total craving scores or those of the three underlying factors.

### Results

For the first CFA model tested, examination of standardized residuals indicated the average absolute standardized residual was 0.044, whereas the off-diagonal absolute standardized residual was 0.050. Furthermore, 91.67% of all standardized residual fell between -0.1 and 0.1, with a further 7.50% occurring between 0.1 and 0.2, indicating a good fit to the model overall. Fit Statistics (S–B χ^2^ = 162.86, *p* < 0.01, RMSEA = 0.056 [CI 0.042 -0.069], CFI = 0.927, NNFI = 0.911, MFI = 0.873) indicated an adequate fit to the hypothesized model. Review of unstandardized individual parameter estimates indicated all individual parameter estimates were significant and that standard errors were within reasonable limits (Jöreskog and Sörborm, 1989). Multivariate LM χ^2^ test indicated one parameter (χ^2^ = 23.52, *p* < 0.01) that would potentially lead to improvements in model specification if it were freely estimated. Specifically, question 13 (“All I want to do right now is go climbing”) which was originally fixed to Factor 1 (urge to climb) might improve the model if it was removed.

The second CFA examined the same model except question 13 was removed as an item from Factor 1(Urge to climb). Overall the model indicated an improved fit. Examination of standardized residuals indicated that the average absolute standardized residual was 0.037, whereas the off-diagonal absolute standardized residual was 0.043. Furthermore, 99.99% of all standardized residual fell between -0.2 and 0.2. Satorra–Bentler Chi-Squared was substantially improved from the previous model (S–B χ^2^ = 113.68, *p* < 0.01), as were the fit indices (RMSEA = 0.044 [CI 0.027 -0.059], CFI = 0.956, NNFI = 0.946, MFI = 0.931). As with the previous model, unstandardized individual parameter estimates indicated that all individual parameter estimates were significant and that standard errors were within reasonable limits.

After controlling for all three covariates, the ANCOVA revealed a significant increase in total craving scores in participants who completed the questionnaire in the presence of climbing related cues [*F*(1,249) = 9.70, *p* = 0.002, $\eta_{p}^{2}$ = 0.04]. However, individually none of the covariates were significantly related to total craving scores (*p* > 0.05). A MANCOVA indicated a significant overall difference across all three sub-factors when taking into account the effects of the three covariates [*F*(3,247) = 4.421, *p* = 0.005, $\eta_{p}^{2}$ = 0.051]. Of the three covariates, only ‘frequency of climbing’ was significantly related to the three measures of craving scores [*F*(3,274) = 5.07, *p* = 0.002, $\eta_{p}^{2}$ = 0.058] with Beta values indicating that as climbing frequency increased, so too did craving via negative (*B* = 0.71, *p* = 0.01) and positive (*B* = 0.21, *p* = 0.47) reinforcement. In contrast, as frequency increased, a significant reduction in “urge to climb” was observed (*B* = -0.09, *p* = 0.01). Follow up principal component analysis indicated one discriminant function that explained 100% of the variance (Canonical *R*^2^ = 0.05). This function significantly differentiated between groups (image vs. no image) [Λ = 0.945, χ^2^(3) = 14.78, *p* = 0.002] with canonical correlation coefficients (negative reinforcement, *r* = 0.931; urge, *r* = 0.51; positive, *r* = 0.302) indicating negative reinforcement contributed the most to separation between those who completed the questionnaire in the presence of images and those who did not (Bray and Maxwell, 1985). Follow up calculations of Cohen’s *d* effect sizes between the image and no image conditions indicated a small to medium effect size for total craving and negative reinforcement, and a small effect size for ‘urge to climb,’ and ‘positive reinforcement’ (Cohen, 1988). See **Table 2**.

**Table 2:** Total RCCQ craving score and mean factor scores by cue condition.

	Cue condition	*M*	*SD*	Cohen’s *d*
Total craving	No image	57.06	6.10	
	Image	59.58	6.83	0.39
Negative reinforcement	No image	3.2915	0.57064	
	Image	3.5664	0.70103	0.42
Urge to climb	No image	4.0659	0.60315	
	Image	4.196	0.5242	0.24
Positive reinforcement	No image	4.2744	0.47108	
	Image	4.3568	0.42622	0.18

Simple regressions for each sub-factor and overall craving indicated that ‘number of days since last climbed’ explained 17% (β = -0.02, *p* = 0.07), 4% (β = -0.004, *p* < 0.01), <1% (β = -0.002, *p* = 0.92), and <1% (β = -0.001, *p* = 0.17) for total craving, ‘urge to climb,’ ‘negative reinforcement,’ and ‘positive reinforcement,’ respectively. Beta values were only significant for ‘urge to climb,’ but negative beta values for all dependents indicated that craving scores generally decreased the longer the absence.

## Overall Discussion

The aim of the studies presented here, was to develop a tool for measuring the state of craving that has previously been identified in rock climbers and mountaineers (Heirene et al., 2016), with the aim of better understanding and quantifying this state. In a two-study development and validation process, support was provided for the factor structure and predictive validity of the Rock Climbing Craving Questionnaire. Specifically, factor analysis revealed a three-factor structure consisting of (*1*) *Urge to climb*, (*2*) *Negative reinforcement*, and (*3*) *Positive reinforcement*. Factor 1 consisted of items reflecting intention to go climbing in the near future and may be considered reminiscent of an urge or craving to go climbing. Factor 2 consisted of items reflecting relief from the negative consequences of not climbing and may be considered reminiscent of climbing-related withdrawal symptoms. Factor 3 consisted of items reflecting a beneficial effect of going climbing and may be considered reminiscent of anticipating an experiential high from going climbing. These factors are comparable to those found in other derivatives of the QSU (e.g., Davies et al., 2000; James et al., 2004; West and Roderique-Davies, 2007) representing desire and intention, positive reinforcement and negative reinforcement. Study 2 confirmed this factor structure.

Given that no other climbing craving questionnaires exist, it was not possible to directly measure construct validity. Instead, we tested the predictive validity (the ability to predict predefined relationships with other variables) of the scale by presenting the questionnaire with and without cues (i.e., climbing related photos) and investigating the relationship between days since last climb (abstinence) and scores on the questionnaire. The craving literature predicts that scores for craving would be higher in the presence of cues and should increase with days since last climb (*see* Roderique-Davies, 2008). The results revealed that for all three factors there were small to medium effect size increases in craving in the presence of climbing-related cues. This matches previous drug/pharmacological research which has shown the presence of relevant cues increases an individual’s craving (e.g., Davies et al., 2000; Goldstein et al., 2009; Schiffer et al., 2009). The highest effect size was observed in the Negative Reinforcement Factor. This is consistent with the findings of Heirene et al. (2016) who reported that rock-climbing athletes appear to experience withdrawal symptoms comparable to individuals with substance and behavioral addictions.

When compared to previous drug research (e.g., McClernon et al., 2009) the lack of positive relationships between time since last climb and craving scores is surprising. Even though the participants in this instance were not drug users, the evidence from extreme sport participants would indicate that abstinence does increase craving (Heirene et al., 2016). West and Roderique-Davies (2007) found a similar relationship between abstinence and craving when they validated the Caffeine Craving Questionnaire. They suggested that as abstinence was not enforced, and participants simply completed the questionnaire without altering their caffeine usage patterns, this abstinence was unlikely to predict craving. In our study, the same is true; although participants displayed a range of abstinence patterning, this abstinence was neither requested nor enforced. From a cognitive-behavioral perspective, individuals who feel they are in control (i.e., have a choice to climb or not) are less likely to experience emotional disturbance (Neenan and Dryden, 2000). Craving is likely to be greatest therefore when individuals are forced to abstain for a period of time and maintain an intention to partake in the activity they crave (e.g., Voglewede and Noel, 2003). However, caution should be taken with this as further studies are needed to clarify the role of abstinence in this context.

### Study Limitations

The present study may be limited by the heterogeneous participant sample, which comprised climbers and mountaineers with multiple primary climbing disciplines, including indoor climbing, outdoor traditional climbing, alpine climbing and ice climbing. Indoor climbing differs considerably from alpine and other outdoor climbing not only in setting, but also in level of risk. Thus, the type and degree of craving could also differ considerably between these sub-disciplines. Additionally, the inclusion of climbers with multiple primary disciplines may have influenced the degree to which the climbing-related cues presented during Study 2 affected craving levels, as these were not tailored to each person’s specific climbing discipline. Future research may wish to recruit climbers of only one primary climbing activity or compare between disciplines in order to better understand how this affects craving and other features of addiction.

### Practical Implications and Directions for Future Research

At this stage, given the preliminary nature of the data, any practical implications are tentative. However, if as shown here, craving for climbing (and potentially other extreme sports) is similar to that experienced by drug-users and addicts, there is the potential climbing and other extreme sports could be used as a replacement therapy for drug users. Specifically, in contrast to drug use, climbing is a socially acceptable activity. Therefore, it is possible that craving experienced by drug users might be lessened/replaced if users are encouraged to participate in climbing activities (or possibly other extreme sports). While to date there is little evidence as to the mechanisms by which these interventions might work, Clough et al. (2016) have recently highlighted several factors associated with adventure sports which may serve as therapeutic components if used as an intervention for mental health issues. These include physical activity as a tool for improving both physical and psychological health; physical and psychological challenge as a method of enhancing mental toughness; and environmental exposure as a means of engendering a sense of well-being and connection with natural environments. More broadly, both traditional sports and exercise routines have shown promise as interventions for drug-addiction, with suggestions that exercise may attenuate craving states and reduce relapse through interactions with the neurotransmitters active in neural reward pathways, including dopamine and glutamate (Lynch et al., 2013). Though, whether the unique characteristics of extreme sports make them more suitable candidates for addiction therapy – as Clough et al. (2016) suggestions would indicate – remains unknown. Thus, the use of extreme sports as an intervention for problematic substance use requires further investigation in order to better understand the potential role these sports could play in a therapeutic context.

It would be of value to investigate the presence of craving in non-extreme sports. To date, investigations of sport-related withdrawal symptoms have been mostly confined to athletes of extreme sports, in line with the popular media conception of such athletes as “adrenaline addicts.” The motivation for developing a measure of extreme sports craving here, as opposed to a more general ‘sports craving’ measure, was underpinned by these studies which have identified conceptual and phenomenological similarities between the experiences of extreme sports athletes and those with an addiction (e.g., Heirene et al., 2016). Nonetheless, it is necessary to determine whether the type of craving measured here is a unique consequence of the ‘extreme’ properties of climbing and similar sports, or can be induced by participation in any sport or physical activity. Indeed, exercise addiction has been proposed as a discrete nosological entity (Berczik et al., 2012) and identified to some degree in multiple populations (e.g., Lichtenstein et al., 2014), suggesting a comparable craving state may be experienced; though investigations of sport-specific addiction appear scant.

The craving measure developed here may be useful in its direct application for understanding the experience of rock climbers and mountaineers. For example, the RCCQ could be used to explore associations between craving levels and subsequent behaviors such as excessive risk-taking or over-training, allowing athletes and coaches an opportunity to identify at-risk individuals. Use of the RCCQ will also allow a greater understanding of extreme sports craving, allowing comparisons of craving levels between sports (both alternative extreme sports and traditional sports) or activities (e.g., drug use) and further investigations of the factors influencing craving levels. Collecting RCCQ scores and physiological data in combination will also enhance the understanding of the physical experiences associated with the addictive-like states observed in these athletes, providing further opportunities for comparing extreme sports craving with the craving observed in other domains such as drug-addiction.

Experimental research is also warranted to further test the predictive validity of the RCCQ model identified here. For example, given that period of abstinence showed no significant positive relationship with RCCQ scores in this study, future researchers should test the influence of forced periods of abstinence on craving scores. In individuals with self-reported exercise addictions, forced periods of abstinence have been associated with decreased mood and vigor, increased tension, anger and confusion, and increased resting heart rate (Aidman and Woollard, 2003), indicating forced abstinence in climbers may be more likely to induce craving states. In climbers and other extreme sports athletes, craving could be investigated during periods of injury in order to circumvent the need to proscribe participation. In addition, more specific research is needed on the influence of cues on craving. In particular, testing the influence of cues in a sample of climbers from only one discipline (e.g., rock-climbing), and ensuring that the cues are ecologically valid and personally meaningful (e.g., familiar locations or familiar climbers). Finally, although research has examined personality factors associated with both substance abusers and extreme sports participants (e.g., Breivik, 1996; Slanger and Rudestam, 1997; Loxton et al., 2008; Roderique-Davies and Shearer, 2010), to date these groups have been examined in isolation of one another. Therefore, research is warranted which compares both groups for both craving and personality (e.g., sensation seeking traits or impulsivity). This type of research will allow a greater understanding of how craving presents in extreme sports such as climbing.

## Conclusion

Overall, the understanding of addiction and craving in an extreme sports setting remains in its infancy compared with the understanding of these concepts in domains such as drug-use and gambling. Nonetheless, the ability to measure and quantify climbing craving, as possible through the RRCQ developed here, will allow a greater understanding of extreme sports craving and how this compares with other forms of craving. The preliminary results obtained here corroborate suggestions that there may be conceptual similarities between extreme sport and drug cravings, including a basis in positive and negative reinforcement and an accentuating influence of cue-exposure.

## Author Contributions

GR-D and DS were involved in all phases of the research, including the design, data collection, analysis, and the writing of the manuscript. RH contributed to the collection and interpretation of data, writing of the manuscript and approving the final version. SM contributed to the interpretation of data, writing of the manuscript and approving the final version. All authors are accountable for all aspects of the work in relation to accuracy and integrity.

## Conflict of Interest Statement

The authors declare that the research was conducted in the absence of any commercial or financial relationships that could be construed as a potential conflict of interest.

## References

[B1] Aidman E. V. Woollard S. (2003). The influence of self-reported exercise addiction on acute emotional and physiological responses to brief exercise deprivation. *Psychol. Sport Exerc.* 4 225–236. 10.1016/S1469-0292(02)00003-1

[B2] Bentler P. M. (2005). *EQS 6 Structural Equation Program Manual.* Los Angeles, CA: Multivariate Software.

[B3] Bentler P. M. Bonett D. G. (1980). Significance tests and goodness-of-fit in the analysis of covariance structures. *Psychol. Bull.* 88 588–600. 10.1037/0033-2909.88.3.588

[B4] Bentler P. M. Dijkstra T. (1985). “Efficient estimation via linearization in structural models”, in *Multivariate Analysis* ed. Krishnaiah P. R. (Amsterdam: North Holland) 9–42.

[B5] Berczik K. Szabó A. Griffiths M. D. Kurimay T. Kun B. Urbán R. (2012). Exercise addiction: symptoms, diagnosis, epidemiology, and etiology. *Subst. Use Misuse* 47 403–417. 10.3109/10826084.2011.639120 22216780

[B6] Biddle S. J. H. Markland D. Gilbourne D. Chatzisarantis N. L. D. Sparkes A. C. (2001). Research methods in sport and exercise psychology: quantitative and qualitative issues. *J. Sports Sci.* 19 777–809. 10.1080/026404101317015438 11561674

[B7] Billieux J. Schimmenti A. Khazaal Y. Maurage P. Heeren A. (2015). Are we overpathologizing everyday life? A tenable blueprint for behavioral addiction research. *J. Behav. Addict.* 4 119–123. 10.1556/2006.4.2015.009 26014667 PMC4627665

[B8] Bray J. H. Maxwell S. E. (1985). *Multivariate Analysis of Variance (No. 54).* London: Sage 10.4135/9781412985222

[B9] Breivik G. (1996). Personality, sensation seeking and risk taking among Everest climbers. *Int. J. Sport Psychol.* 27 308–320.

[B10] Brymer E. Schweitzer R. (2013). The search for freedom in extreme sports: a phenomenological exploration. *Psychol. Sport and Exerc.* 14 865–873. 10.1016/j.psychsport.2013.07.004

[B11] Buckley R. C. (2015). Adventure thrills are addictive. *Front. Psychol.* 6:1915. 10.3389/fpsyg.2015.01915 26733908 PMC4679910

[B12] Byrne B. M. (2008). *Structural Equation Modeling with EQS: Basic Concepts, Applications, and Programming.* London: Routledge.

[B13] Clark D. (1994). Craving for alcohol. *J. Psychopharmacol.* 9:73.

[B14] Clough P. Mackenzie H. S. Mallabon L. Brymer E. (2016). Adventurous physical activity environments: a mainstream intervention for mental health. *Sports Med.* 46 963–968. 10.1007/s40279-016-0503-3 26895993

[B15] Cohen J. (eds) (1988). “The effect size index: d,” in *Statistical Power Analysis for the Behavioural Sciences* (Cambridge, MA: Academic Press)

[B16] Davies G. Morgan M. Willner P. (2000). Smoking-related cues elicit craving in tobacco chippers: a replication and validation of the two-factor structure of the questionnaire of smoking urges. *Psychopharmacology* 152 334–342. 10.1007/s002130000526 11105944

[B17] Diehm R. Armatas C. (2004). Surfing: an avenue for socially acceptable risk-taking, satisfying needs seeking for sensation seeking experience. *Personal. Individ. Differ.* 36 663–677. 10.1016/S0191-8869(03)00124-7

[B18] Field A. (2013). *Discovering Statistics using IBM SPSS Statistics.* London: Sage.

[B19] Franken I. H. A. Zijlstra C. Muris P. (2006). Are nonpharmacological induced rewards related to anhedonia? A study among skydivers. *Prog. NeuroPsychopharmacol. Biol. Psychiatry* 30 297–300. 10.1016/j.pnpbp.2005.10.011 16303225

[B20] Goldstein R. Z. Tomasi D. Alia-Klein N. Carillo J. H. Maloney T. Woicik P. A. (2009). Dopaminergic response to drug words in cocaine addiction. *J. Neurosci.* 29 6001–6006. 10.1523/JNEUROSCI.4247-08.2009 19420266 PMC2691777

[B21] Heirene R. M. Shearer D. Roderique-Davies G. Mellalieu S. D. (2016). Addiction in extreme sports: an exploration of withdrawal states in rock climbers. *J. Behav. Addict.* 5 332–341. 10.1556/2006.5.2016.039 27348554 PMC5387785

[B22] Hendrickson A. E. White P. O. (1964). PROMAX: a quick method for rotation to oblique simple structure. *Br. J. Math. Stat. Psychol.* 17 65–70. 10.1111/j.2044-8317.1964.tb00244.x

[B23] Hu L. T. Bentler P. M. (1999). Cutoff criteria for fit indexes in covariance structure analysis: conventional criteria versus new alternatives. *Struct. Equ. Modeling* 6 1–55. 10.1080/10705519909540118

[B24] James D. Davies G. Willner P. (2004). The development and initial validation of a questionnaire to measure craving for amphetamine. *Addiction* 99 1181–1188. 10.1111/j.1360-0443.2004.00819.x 15317639

[B25] Jöreskog K. G. Sörborm D. (1989). *LISREL 7 user’s Reference Guide.* Chicago, IL: Scientific Software International.

[B26] Kerr J. H. Mackenzie H. S. (2012). Multiple motives for participating in adventure sports. *Psychol. Sport Exerc.* 13 649–657. 10.1016/j.psychsport.2012.04.002

[B27] Kreusch F. Billieux J. Quertemont E. (2017). Alcohol-cue exposure decreases response inhibition towards alcohol-related stimuli in detoxified alcohol-dependent patients. *Psychiatry Res.* 249 232–239. 10.1016/j.psychres.2017.01.019 28126578

[B28] Lichtenstein M. B. Larsen K. S. Christiansen E. Støving R. K. Bredahl T. V. G. (2014). Exercise addiction in team sport and individual sport: prevalences and validation of the exercise addiction inventory. *Addict. Res. Theory* 22 431–437. 10.3109/16066359.2013.875537

[B29] Love A. James D. Willner P. (1998). A comparison of two alcohol craving questionnaires. *Addiction* 93 1091–1102. 10.1046/j.1360-0443.1998.937109113.x 9744139

[B30] Loxton N. J. Wan V. L. N. Ho A. M. C. Cheung B. K. L. Tam N. Leung F. Y. K. (2008). Impulsivity in Hong Kong-Chinese club-drug users. *Drug Alcohol Depend.* 95 81–89. 10.1016/j.drugalcdep.2007.12.009 18242007

[B31] Luttenberger K. Stelzer E.-M. Först S. Schopper M. Kornhuber J. Book S. (2015). Indoor rock climbing (bouldering) as a new treatment for depression: study design of a waitlist-controlled randomized group pilot study and the first results. *BMC Psychiatry* 15:201. 10.1186/s12888-015-0585-8 26302900 PMC4548691

[B32] Lynch W. J. Peterson A. B. Sanchez V. Abel J. Smith M. A. (2013). Exercise as a novel treatment for drug addiction: a neurobiological and stage-dependent hypothesis. *Neurosci. Biobehav. Rev.* 37 1622–1644. 10.1016/j.neubiorev.2013.06.011 23806439 PMC3788047

[B33] Mardia K. V. (1970). Measures of multivariate skewness and kurtosis with applications. *Biometrika* 57 519–530. 10.2307/2334770

[B34] Mardia K. V. (1974). Applications of some measures of multivariate skewness and kurtosis in testing normality and robustness studies. *Sankhya Ind. J. Stat. Ser. B* 115–128.

[B35] McClernon F. J. Kozinck R. V. Lutz A. M. Rose J. E. (2009). 24-h smoking abstinence potentiates fMRI-BOLD activation to smoking cues in cerebral cortex and dorsal striatum. *Psychopharmacology* 204 25–35. 10.1007/s00213-008-1436-9 19107465 PMC2810714

[B36] McDonald R. P. (1989). An index of goodness-of-fit based on noncentrality. *J. Classif.* 6 97–103. 10.1007/BF01908590 24764609

[B37] Neenan M. Dryden W. (2000). *Essential Cognitive Therapy.* London: Whurr Publishers.

[B38] Price I. R. Bundesen C. (2005). Emotional changes in skydivers in relation to experience. *Personal. Individ. Differ.* 38 1203–1211. 10.1016/j.paid.2004.08.003

[B39] Roderique-Davies G. (2008). “Cigarette craving: exploring the enigma,” in *Smoking Cessation: Theory, Interventions and Prevention*, ed. Landow J. E. (NewYork, NY: Nova Science Publishers), 255–282.

[B40] Roderique-Davies G. Shearer D. (2010). Estimated lifetime drug use, impulsivity and psychopathology in recreational ecstasy users. *J. Subst. Use* 15 215–225. 10.3109/14659890903271616

[B41] Satorra A. Bentler P. M. (1994). “Corrections to test statistics and standard errors in covariance structure analysis,” in *Latent Variable Analysis: Applications for Developmental Research* eds Eye A. von Clogg C. C. (Thousand Oaks, CA: Sage) 339–419.

[B42] Schiffer W. K. Liebling C. N. B. Reiszel C. Hooker J. M. Brodie J. D. Dewey S. L. (2009). Cue-induced dopamine release predicts cocaine preference: positron emission tomography studies in freely moving rodents. *J. Neurosci.* 29 6176–6185. 10.1523/JNEUROSCI.5221-08.2009 19439595 PMC6665516

[B43] Shen W. Liu Y. Li L. Zhang Y. Zhou W. (2012). Negative moods correlate with craving in female methamphetamine users enrolled in compulsory detoxification. *Subst. Abuse Treat. Prev. Policy* 7 44–44. 10.1186/1747-597X-7-44 23110820 PMC3551715

[B44] Singleton E. G. Tiffany S. T. Henningfield J. E. (2004). “The alcohol craving QUESTIONNAIRE (ACQ-Now),” in *Assessing Alcohol Problems: A Guide for Clinicians and Researchers* 2nd Edn, eds Allen J. P. Wilson V. B. (Bethesda, MD: National Institute on Alcohol Abuse and Alcoholism) 271–281.

[B45] Slanger E. Rudestam K. E. (1997). Motivation and disinhibition in high risk sports: sensation seeking and self-efficacy. *J. Res. Personal.* 31 355–374. 10.1006/jrpe.1997.2193

[B46] Steiger J. H. Lind J. C. (1980). “May, statistically based tests for the number of factors. *Paper Presented at the Annual Meeting of the Psychometric Society* Iowa City, IA.

[B47] Tiffany S. T. (1990). A cognitive model of drug urges and drug-use behavior: role of automatic and non-automatic processes. *Psychol. Rev.* 97 147–168. 10.1037/0033-295X.97.2.147 2186423

[B48] Tiffany S. T. Drobes D. J. (1991). The development and initial validation of a questionnaire on smoking urges. *Br. J. Addict.* 86 1467–1476. 10.1111/j.1360-0443.1991.tb01732.x 1777741

[B49] Tiffany S. T. Singleton E. Haertzen C. A. Henningfield J. E. (1993). The development of a cocaine craving questionnaire. *Drug Alcohol Depend.* 34 19–28. 10.1016/0376-8716(93)90042-O 8174499

[B50] Tiffany S. T. Wray J. M. (2012). The clinical significance of drug craving. *Ann. N. Y. Acad. Sci. U.S.A*. 1248 1–17. 10.1111/j.1749-6632.2011.06298.x 22172057 PMC4041083

[B51] Tucker L. R. Lewis C. (1973). A reliability coefficient for maximum likelihood factor analysis. *Psychometrika* 38 1–10. 10.1007/BF02291170

[B52] UNDCP and WHO (1992). *“Informal Expert Committee on the Craving Mechanism”: Report.* Technical Report Series No. 92-54439T Geneva: United Nations International Drug Control Programme and World Health Organization.

[B53] Voglewede J. P. Noel M. E. (2003). Predictors of current need to smoke in inmates of a smoke-free jail. *Addict. Behav.* 29 343–348. 10.1016/j.addbeh.2003.08.048 14732422

[B54] West O. Roderique-Davies G. (2007). Development and initial validation of a caffeine craving questionnaire. *J. Psychopharmacol.* 22 80–91. 10.1177/0269881107082746 18187535

[B55] Woodman T. Hardy L. Barlow M. Le Scanff C. (2010). Motives for participation in prolonged engagement high-risk sports: an agentic emotion regulation perspective. *Psychol. Sport Exerc.* 11 345–352. 10.1016/j.psychsport.2010.04.002

